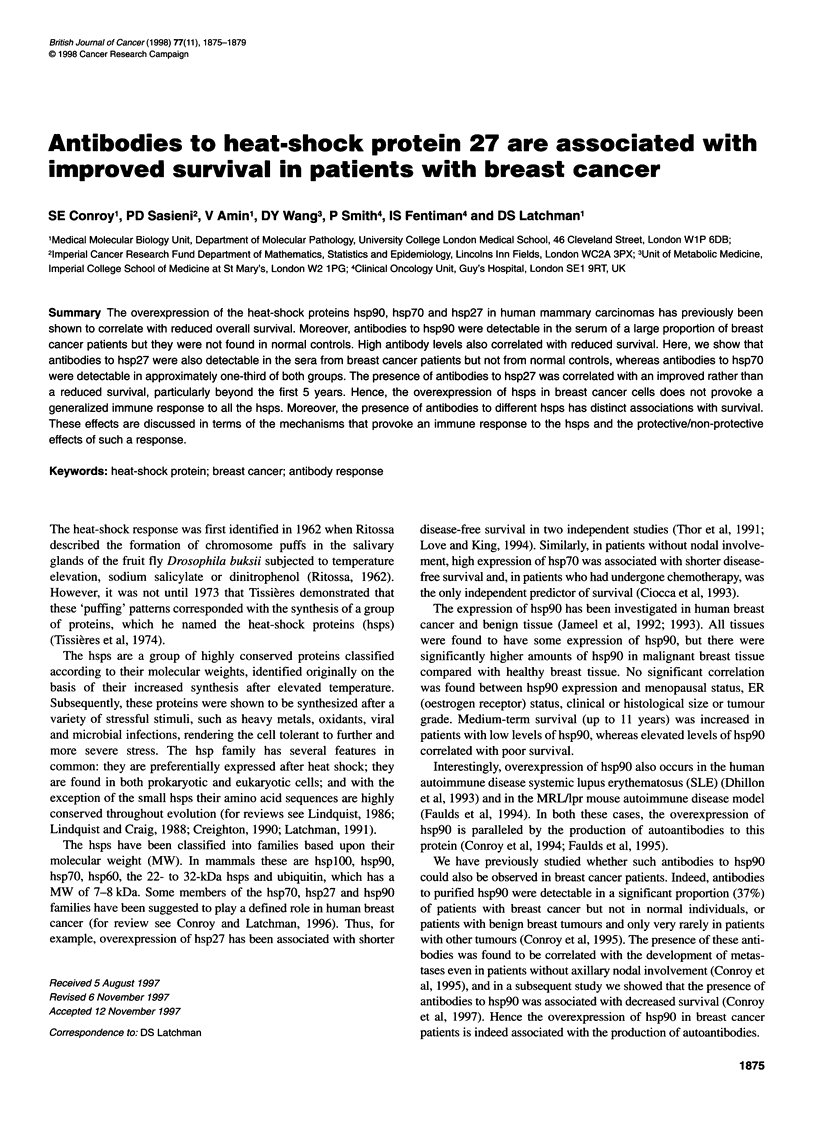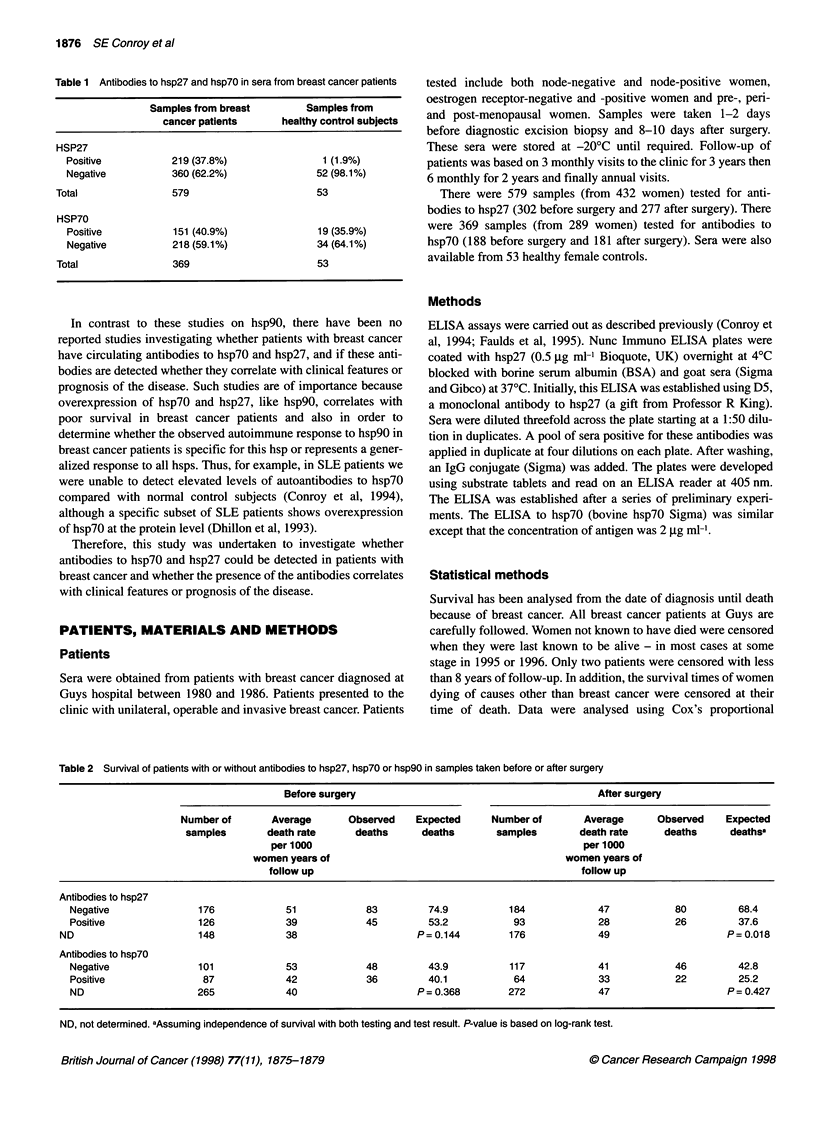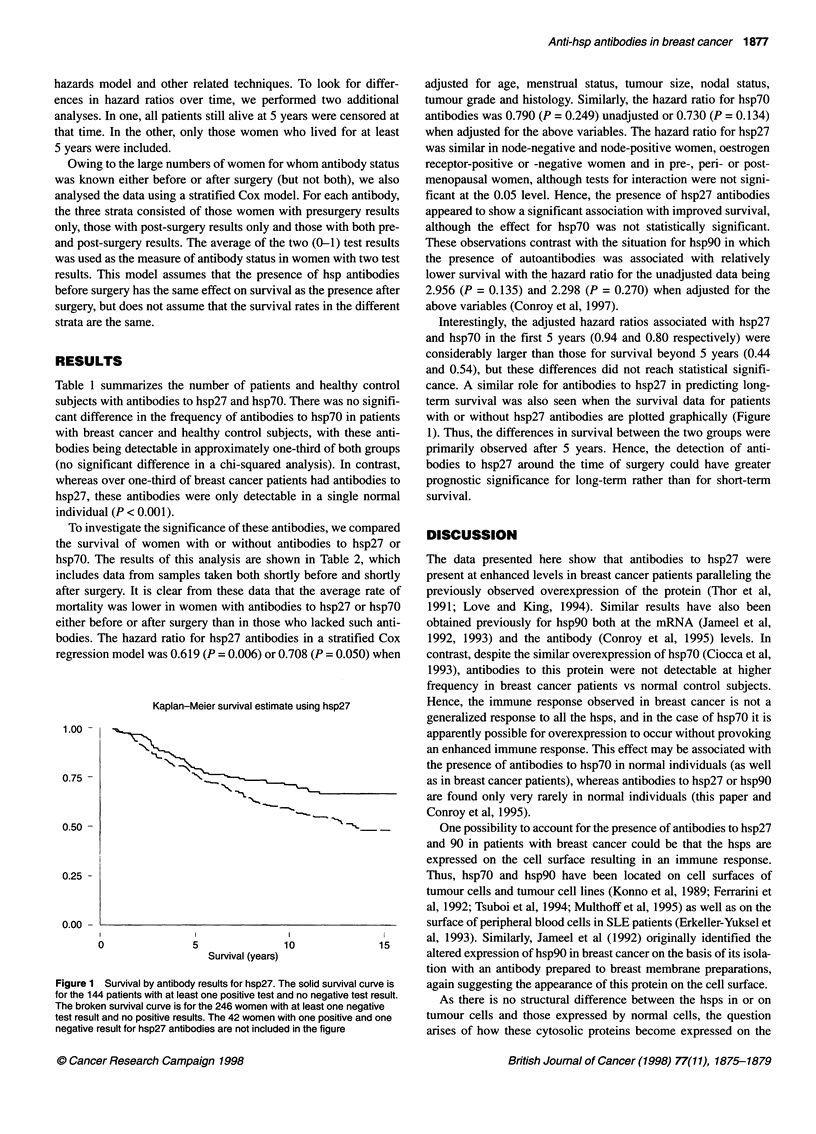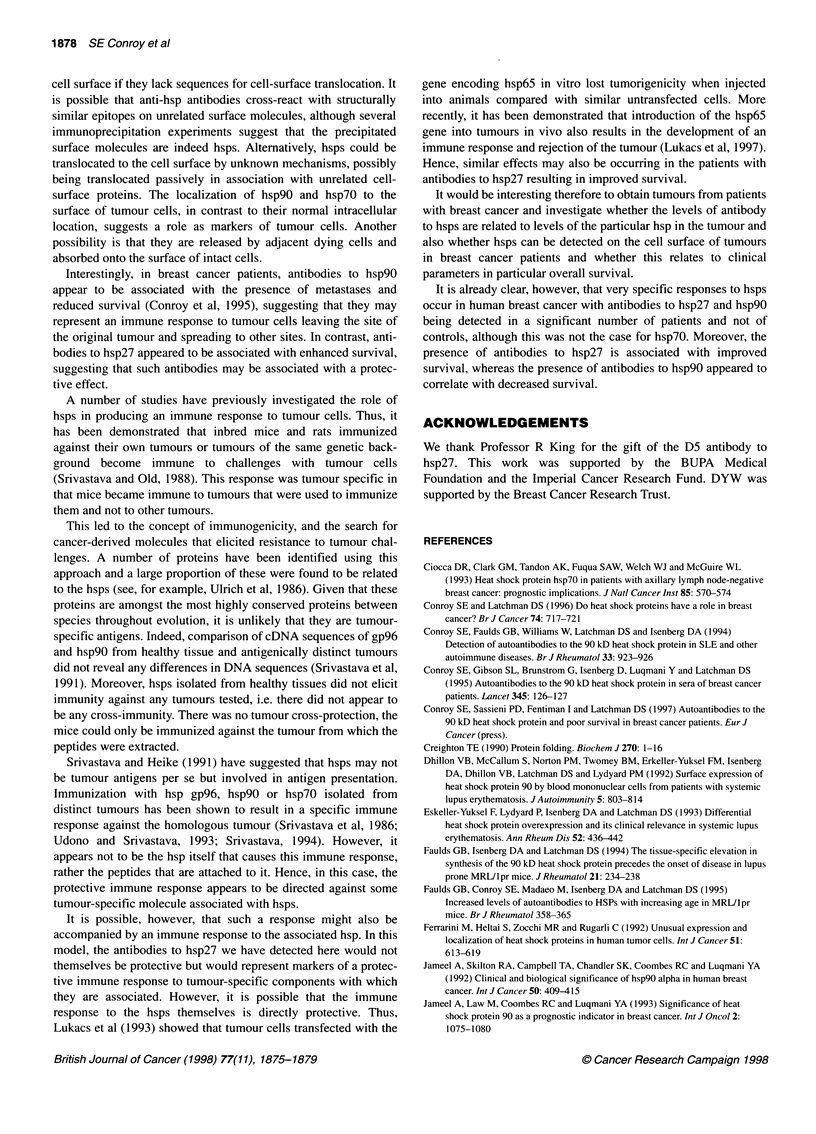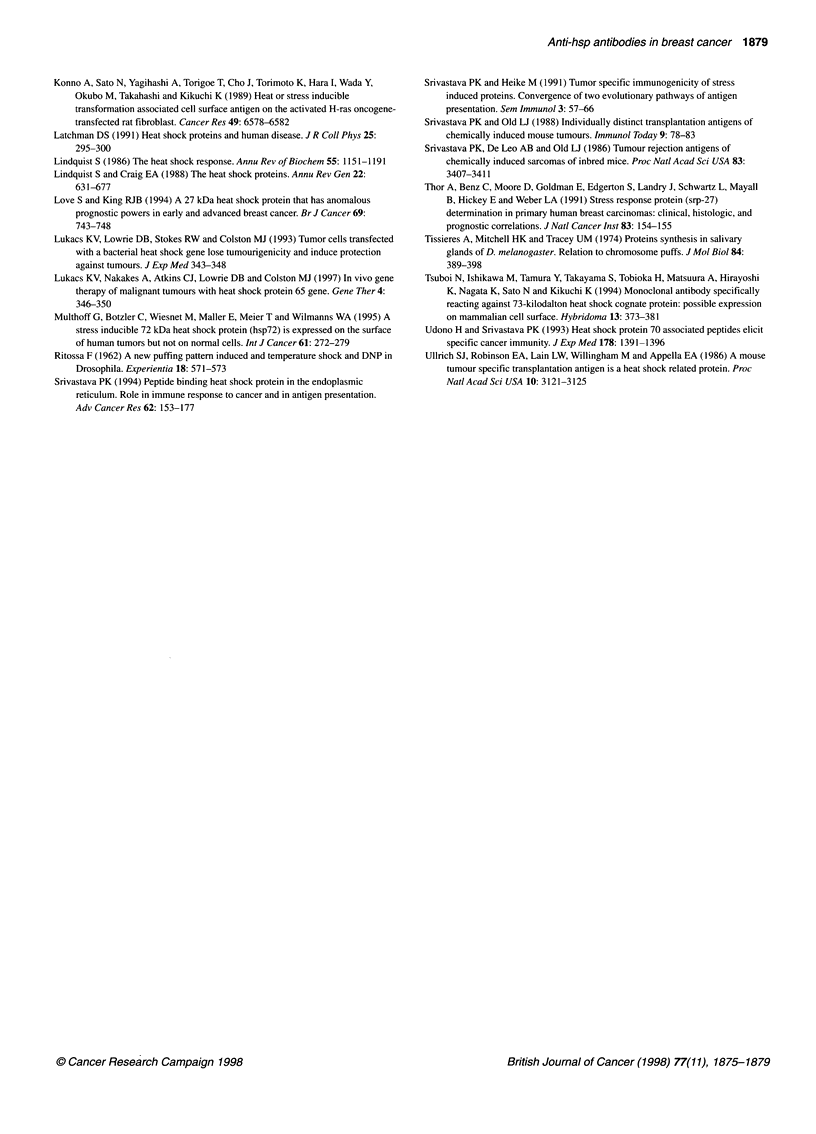# Antibodies to heat-shock protein 27 are associated with improved survival in patients with breast cancer.

**DOI:** 10.1038/bjc.1998.312

**Published:** 1998-06

**Authors:** S. E. Conroy, P. D. Sasieni, V. Amin, D. Y. Wang, P. Smith, I. S. Fentiman, D. S. Latchman

**Affiliations:** Department of Molecular Pathology, University College London Medical School, UK.

## Abstract

The overexpression of the heat-shock proteins hsp90, hsp70 and hsp27 in human mammary carcinomas has previously been shown to correlate with reduced overall survival. Moreover, antibodies to hsp90 were detectable in the serum of a large proportion of breast cancer patients but they were not found in normal controls. High antibody levels also correlated with reduced survival. Here, we show that antibodies to hsp27 were also detectable in the sera from breast cancer patients but not from normal controls, whereas antibodies to hsp70 were detectable in approximately one-third of both groups. The presence of antibodies to hsp27 was correlated with an improved rather than a reduced survival, particularly beyond the first 5 years. Hence, the overexpression of hsps in breast cancer cells does not provoke a generalized immune response to all the hsps. Moreover, the presence of antibodies to different hsps has distinct associations with survival. These effects are discussed in terms of the mechanisms that provoke an immune response to the hsps and the protective/non-protective effects of such a response.


British Journal of Cancer (1998) 77(11), 1875–1879
© 1998 Cancer Research Campaign

### Antibodies to heat-shock protein 27 are associated with improved survival in patients with breast cancer

**SE Conroy[1], PD Sasieni[2], V Amin[1], DY Wang[3], P Smith[4], IS Fentiman[4] and DS Latchman[1]**

[1]Medical Molecular Biology Unit, Department of Molecular Pathology, University College London Medical School, 46 Cleveland Street, London W1P 6DB; [2]Imperial Cancer Research Fund Department of Mathematics, Statistics and Epidemiology, Lincolns Inn Fields, London WC2A 3PX; [3]Unit of Metabolic Medicine, Imperial College School of Medicine at St Mary's, London W2 1PG; [4]Clinical Oncology Unit, Guy's Hospital, London SE1 9RT, UK

**Summary** The overexpression of the heat-shock proteins hsp90, hsp70 and hsp27 in human mammary carcinomas has previously been shown to correlate with reduced overall survival. Moreover, antibodies to hsp90 were detectable in the serum of a large proportion of breast cancer patients but they were not found in normal controls. High antibody levels also correlated with reduced survival. Here, we show that antibodies to hsp27 were also detectable in the sera from breast cancer patients but not from normal controls, whereas antibodies to hsp70 were detectable in approximately one-third of both groups. The presence of antibodies to hsp27 was correlated with an improved rather than a reduced survival, particularly beyond the first 5 years. Hence, the overexpression of hsps in breast cancer cells does not provoke a generalized immune response to all the hsps. Moreover, the presence of antibodies to different hsps has distinct associations with survival. These effects are discussed in terms of the mechanisms that provoke an immune response to the hsps and the protective/non-protective effects of such a response.

**Keywords:** heat-shock protein; breast cancer; antibody response

The heat-shock response was first identified in 1962 when Ritossa described the formation of chromosome puffs in the salivary glands of the fruit fly *Drosophila buksii* subjected to temperature elevation, sodium salicylate or dinitrophenol (Ritossa, 1962). However, it was not until 1973 that Tissières demonstrated that these 'puffing' patterns corresponded with the synthesis of a group of proteins, which he named the heat-shock proteins (hsps) (Tissières et al, 1974).

The hsps are a group of highly conserved proteins classified according to their molecular weights, identified originally on the basis of their increased synthesis after elevated temperature. Subsequently, these proteins were shown to be synthesized after a variety of stressful stimuli, such as heavy metals, oxidants, viral and microbial infections, rendering the cell tolerant to further and more severe stress. The hsp family has several features in common: they are preferentially expressed after heat shock; they are found in both prokaryotic and eukaryotic cells; and with the exception of the small hsps their amino acid sequences are highly conserved throughout evolution (for reviews see Lindquist, 1986; Lindquist and Craig, 1988; Creighton, 1990; Latchman, 1991).

The hsps have been classified into families based upon their molecular weight (MW). In mammals these are hsp100, hsp90, hsp70, hsp60, the 22- to 32-kDa hsps and ubiquitin, which has a MW of 7–8 kDa. Some members of the hsp70, hsp27 and hsp90 families have been suggested to play a defined role in human breast cancer (for review see Conroy and Latchman, 1996). Thus, for example, overexpression of hsp27 has been associated with shorter disease-free survival in two independent studies (Thor et al, 1991; Love and King, 1994). Similarly, in patients without nodal involvement, high expression of hsp70 was associated with shorter disease-free survival and, in patients who had undergone chemotherapy, was the only independent predictor of survival (Ciocca et al, 1993).

The expression of hsp90 has been investigated in human breast cancer and benign tissue (Jameel et al, 1992; 1993). All tissues were found to have some expression of hsp90, but there were significantly higher amounts of hsp90 in malignant breast tissue compared with healthy breast tissue. No significant correlation was found between hsp90 expression and menopausal status, ER (oestrogen receptor) status, clinical or histological size or tumour grade. Medium-term survival (up to 11 years) was increased in patients with low levels of hsp90, whereas elevated levels of hsp90 correlated with poor survival.

Interestingly, overexpression of hsp90 also occurs in the human autoimmune disease systemic lupus erythematosus (SLE) (Dhillon et al, 1993) and in the MRL/lpr mouse autoimmune disease model (Faulds et al, 1994). In both these cases, the overexpression of hsp90 is paralleled by the production of autoantibodies to this protein (Conroy et al, 1994; Faulds et al, 1995).

We have previously studied whether such antibodies to hsp90 could also be observed in breast cancer patients. Indeed, antibodies to purified hsp90 were detectable in a significant proportion (37%) of patients with breast cancer but not in normal individuals, or patients with benign breast tumours and only very rarely in patients with other tumours (Conroy et al, 1995). The presence of these antibodies was found to be correlated with the development of metastases even in patients without axillary nodal involvement (Conroy et al, 1995), and in a subsequent study we showed that the presence of antibodies to hsp90 was associated with decreased survival (Conroy et al, 1997). Hence the overexpression of hsp90 in breast cancer patients is indeed associated with the production of autoantibodies.

*Received 5 August 1997*
*Revised 6 November 1997*
*Accepted 12 November 1997*

*Correspondence to:* DS Latchman

**Table 1** Antibodies to hsp27 and hsp70 in sera from breast cancer patients

| | Samples from breast cancer patients | Samples from healthy control subjects |
|---|---|---|
| HSP27 | | |
| Positive | 219 (37.8%) | 1 (1.9%) |
| Negative | 360 (62.2%) | 52 (98.1%) |
| Total | 579 | 53 |
| HSP70 | | |
| Positive | 151 (40.9%) | 19 (35.9%) |
| Negative | 218 (59.1%) | 34 (64.1%) |
| Total | 369 | 53 |

In contrast to these studies on hsp90, there have been no reported studies investigating whether patients with breast cancer have circulating antibodies to hsp70 and hsp27, and if these antibodies are detected whether they correlate with clinical features or prognosis of the disease. Such studies are of importance because overexpression of hsp70 and hsp27, like hsp90, correlates with poor survival in breast cancer patients and also in order to determine whether the observed autoimmune response to hsp90 in breast cancer patients is specific for this hsp or represents a generalized response to all hsps. Thus, for example, in SLE patients we were unable to detect elevated levels of autoantibodies to hsp70 compared with normal control subjects (Conroy et al, 1994), although a specific subset of SLE patients shows overexpression of hsp70 at the protein level (Dhillon et al, 1993).

Therefore, this study was undertaken to investigate whether antibodies to hsp70 and hsp27 could be detected in patients with breast cancer and whether the presence of the antibodies correlates with clinical features or prognosis of the disease.

### PATIENTS, MATERIALS AND METHODS

### Patients

Sera were obtained from patients with breast cancer diagnosed at Guys hospital between 1980 and 1986. Patients presented to the clinic with unilateral, operable and invasive breast cancer. Patients tested include both node-negative and node-positive women, oestrogen receptor-negative and -positive women and pre-, peri- and post-menopausal women. Samples were taken 1–2 days before diagnostic excision biopsy and 8–10 days after surgery. These sera were stored at –20°C until required. Follow-up of patients was based on 3 monthly visits to the clinic for 3 years then 6 monthly for 2 years and finally annual visits.

There were 579 samples (from 432 women) tested for antibodies to hsp27 (302 before surgery and 277 after surgery). There were 369 samples (from 289 women) tested for antibodies to hsp70 (188 before surgery and 181 after surgery). Sera were also available from 53 healthy female controls.

### Methods

ELISA assays were carried out as described previously (Conroy et al, 1994; Faulds et al, 1995). Nunc Immuno ELISA plates were coated with hsp27 (0.5 μg $ml^{-1}$ Bioquote, UK) overnight at 4°C blocked with borine serum albumin (BSA) and goat sera (Sigma and Gibco) at 37°C. Initially, this ELISA was established using D5, a monoclonal antibody to hsp27 (a gift from Professor R King). Sera were diluted threefold across the plate starting at a 1:50 dilution in duplicates. A pool of sera positive for these antibodies was applied in duplicate at four dilutions on each plate. After washing, an IgG conjugate (Sigma) was added. The plates were developed using substrate tablets and read on an ELISA reader at 405 nm. The ELISA was established after a series of preliminary experiments. The ELISA to hsp70 (bovine hsp70 Sigma) was similar except that the concentration of antigen was 2 μg $ml^{-1}$.

### Statistical methods

Survival has been analysed from the date of diagnosis until death because of breast cancer. All breast cancer patients at Guys are carefully followed. Women not known to have died were censored when they were last known to be alive – in most cases at some stage in 1995 or 1996. Only two patients were censored with less than 8 years of follow-up. In addition, the survival times of women dying of causes other than breast cancer were censored at their time of death. Data were analysed using Cox's proportional

**Table 2** Survival of patients with or without antibodies to hsp27, hsp70 or hsp90 in samples taken before or after surgery

| | Before surgery | | | | After surgery | | | |
|---|---|---|---|---|---|---|---|---|
| | Number of samples | Average death rate per 1000 women years of follow up | Observed deaths | Expected deaths | Number of samples | Average death rate per 1000 women years of follow up | Observed deaths | Expected deaths[a] |
| Antibodies to hsp27 | | | | | | | | |
| Negative | 176 | 51 | 83 | 74.9 | 184 | 47 | 80 | 68.4 |
| Positive | 126 | 39 | 45 | 53.2 | 93 | 28 | 26 | 37.6 |
| ND | 148 | 38 | | $P = 0.144$ | 176 | 49 | | $P = 0.018$ |
| Antibodies to hsp70 | | | | | | | | |
| Negative | 101 | 53 | 48 | 43.9 | 117 | 41 | 46 | 42.8 |
| Positive | 87 | 42 | 36 | 40.1 | 64 | 33 | 22 | 25.2 |
| ND | 265 | 40 | | $P = 0.368$ | 272 | 47 | | $P = 0.427$ |

ND, not determined. [a]Assuming independence of survival with both testing and test result. *P*-value is based on log-rank test.

© Cancer Research Campaign 1998

hazards model and other related techniques. To look for differences in hazard ratios over time, we performed two additional analyses. In one, all patients still alive at 5 years were censored at that time. In the other, only those women who lived for at least 5 years were included.

Owing to the large numbers of women for whom antibody status was known either before or after surgery (but not both), we also analysed the data using a stratified Cox model. For each antibody, the three strata consisted of those women with presurgery results only, those with post-surgery results only and those with both pre- and post-surgery results. The average of the two (0–1) test results was used as the measure of antibody status in women with two test results. This model assumes that the presence of hsp antibodies before surgery has the same effect on survival as the presence after surgery, but does not assume that the survival rates in the different strata are the same.

### RESULTS

Table 1 summarizes the number of patients and healthy control subjects with antibodies to hsp27 and hsp70. There was no significant difference in the frequency of antibodies to hsp70 in patients with breast cancer and healthy control subjects, with these antibodies being detectable in approximately one-third of both groups (no significant difference in a chi-squared analysis). In contrast, whereas over one-third of breast cancer patients had antibodies to hsp27, these antibodies were only detectable in a single normal individual ($P < 0.001$).

To investigate the significance of these antibodies, we compared the survival of women with or without antibodies to hsp27 or hsp70. The results of this analysis are shown in Table 2, which includes data from samples taken both shortly before and shortly after surgery. It is clear from these data that the average rate of mortality was lower in women with antibodies to hsp27 or hsp70 either before or after surgery than in those who lacked such antibodies. The hazard ratio for hsp27 antibodies in a stratified Cox regression model was 0.619 ($P = 0.006$) or 0.708 ($P = 0.050$) when adjusted for age, menstrual status, tumour size, nodal status, tumour grade and histology. Similarly, the hazard ratio for hsp70 antibodies was 0.790 ($P = 0.249$) unadjusted or 0.730 ($P = 0.134$) when adjusted for the above variables. The hazard ratio for hsp27 was similar in node-negative and node-positive women, oestrogen receptor-positive or -negative women and in pre-, peri- or post-menopausal women, although tests for interaction were not significant at the 0.05 level. Hence, the presence of hsp27 antibodies appeared to show a significant association with improved survival, although the effect for hsp70 was not statistically significant. These observations contrast with the situation for hsp90 in which the presence of autoantibodies was associated with relatively lower survival with the hazard ratio for the unadjusted data being 2.956 ($P = 0.135$) and 2.298 ($P = 0.270$) when adjusted for the above variables (Conroy et al, 1997).

Interestingly, the adjusted hazard ratios associated with hsp27 and hsp70 in the first 5 years (0.94 and 0.80 respectively) were considerably larger than those for survival beyond 5 years (0.44 and 0.54), but these differences did not reach statistical significance. A similar role for antibodies to hsp27 in predicting long-term survival was also seen when the survival data for patients with or without hsp27 antibodies are plotted graphically (Figure 1). Thus, the differences in survival between the two groups were primarily observed after 5 years. Hence, the detection of antibodies to hsp27 around the time of surgery could have greater prognostic significance for long-term rather than for short-term survival.

**Figure 1** Survival by antibody results for hsp27. The solid survival curve is for the 144 patients with at least one positive test and no negative test result. The broken survival curve is for the 246 women with at least one negative test result and no positive results. The 42 women with one positive and one negative result for hsp27 antibodies are not included in the figure

### DISCUSSION

The data presented here show that antibodies to hsp27 were present at enhanced levels in breast cancer patients paralleling the previously observed overexpression of the protein (Thor et al, 1991; Love and King, 1994). Similar results have also been obtained previously for hsp90 both at the mRNA (Jameel et al, 1992, 1993) and the antibody (Conroy et al, 1995) levels. In contrast, despite the similar overexpression of hsp70 (Ciocca et al, 1993), antibodies to this protein were not detectable at higher frequency in breast cancer patients vs normal control subjects. Hence, the immune response observed in breast cancer is not a generalized response to all the hsps, and in the case of hsp70 it is apparently possible for overexpression to occur without provoking an enhanced immune response. This effect may be associated with the presence of antibodies to hsp70 in normal individuals (as well as in breast cancer patients), whereas antibodies to hsp27 or hsp90 are found only very rarely in normal individuals (this paper and Conroy et al, 1995).

One possibility to account for the presence of antibodies to hsp27 and 90 in patients with breast cancer could be that the hsps are expressed on the cell surface resulting in an immune response. Thus, hsp70 and hsp90 have been located on cell surfaces of tumour cells and tumour cell lines (Konno et al, 1989; Ferrarini et al, 1992; Tsuboi et al, 1994; Multhoff et al, 1995) as well as on the surface of peripheral blood cells in SLE patients (Erkeller-Yuksel et al, 1993). Similarly, Jameel et al (1992) originally identified the altered expression of hsp90 in breast cancer on the basis of its isolation with an antibody prepared to breast membrane preparations, again suggesting the appearance of this protein on the cell surface.

As there is no structural difference between the hsps in or on tumour cells and those expressed by normal cells, the question arises of how these cytosolic proteins become expressed on the

© Cancer Research Campaign 1998

cell surface if they lack sequences for cell-surface translocation. It is possible that anti-hsp antibodies cross-react with structurally similar epitopes on unrelated surface molecules, although several immunoprecipitation experiments suggest that the precipitated surface molecules are indeed hsps. Alternatively, hsps could be translocated to the cell surface by unknown mechanisms, possibly being translocated passively in association with unrelated cell-surface proteins. The localization of hsp90 and hsp70 to the surface of tumour cells, in contrast to their normal intracellular location, suggests a role as markers of tumour cells. Another possibility is that they are released by adjacent dying cells and absorbed onto the surface of intact cells.

Interestingly, in breast cancer patients, antibodies to hsp90 appear to be associated with the presence of metastases and reduced survival (Conroy et al, 1995), suggesting that they may represent an immune response to tumour cells leaving the site of the original tumour and spreading to other sites. In contrast, antibodies to hsp27 appeared to be associated with enhanced survival, suggesting that such antibodies may be associated with a protective effect.

A number of studies have previously investigated the role of hsps in producing an immune response to tumour cells. Thus, it has been demonstrated that inbred mice and rats immunized against their own tumours or tumours of the same genetic background become immune to challenges with tumour cells (Srivastava and Old, 1988). This response was tumour specific in that mice became immune to tumours that were used to immunize them and not to other tumours.

This led to the concept of immunogenicity, and the search for cancer-derived molecules that elicited resistance to tumour challenges. A number of proteins have been identified using this approach and a large proportion of these were found to be related to the hsps (see, for example, Ulrich et al, 1986). Given that these proteins are amongst the most highly conserved proteins between species throughout evolution, it is unlikely that they are tumour-specific antigens. Indeed, comparison of cDNA sequences of gp96 and hsp90 from healthy tissue and antigenically distinct tumours did not reveal any differences in DNA sequences (Srivastava et al, 1991). Moreover, hsps isolated from healthy tissues did not elicit immunity against any tumours tested, i.e. there did not appear to be any cross-immunity. There was no tumour cross-protection, the mice could only be immunized against the tumour from which the peptides were extracted.

Srivastava and Heike (1991) have suggested that hsps may not be tumour antigens per se but involved in antigen presentation. Immunization with hsp gp96, hsp90 or hsp70 isolated from distinct tumours has been shown to result in a specific immune response against the homologous tumour (Srivastava et al, 1986; Udono and Srivastava, 1993; Srivastava, 1994). However, it appears not to be the hsp itself that causes this immune response, rather the peptides that are attached to it. Hence, in this case, the protective immune response appears to be directed against some tumour-specific molecule associated with hsps.

It is possible, however, that such a response might also be accompanied by an immune response to the associated hsp. In this model, the antibodies to hsp27 we have detected here would not themselves be protective but would represent markers of a protective immune response to tumour-specific components with which they are associated. However, it is possible that the immune response to the hsps themselves is directly protective. Thus, Lukacs et al (1993) showed that tumour cells transfected with the gene encoding hsp65 in vitro lost tumorigenicity when injected into animals compared with similar untransfected cells. More recently, it has been demonstrated that introduction of the hsp65 gene into tumours in vivo also results in the development of an immune response and rejection of the tumour (Lukacs et al, 1997). Hence, similar effects may also be occurring in the patients with antibodies to hsp27 resulting in improved survival.

It would be interesting therefore to obtain tumours from patients with breast cancer and investigate whether the levels of antibody to hsps are related to levels of the particular hsp in the tumour and also whether hsps can be detected on the cell surface of tumours in breast cancer patients and whether this relates to clinical parameters in particular overall survival.

It is already clear, however, that very specific responses to hsps occur in human breast cancer with antibodies to hsp27 and hsp90 being detected in a significant number of patients and not of controls, although this was not the case for hsp70. Moreover, the presence of antibodies to hsp27 is associated with improved survival, whereas the presence of antibodies to hsp90 appeared to correlate with decreased survival.

### ACKNOWLEDGEMENTS

We thank Professor R King for the gift of the D5 antibody to hsp27. This work was supported by the BUPA Medical Foundation and the Imperial Cancer Research Fund. DYW was supported by the Breast Cancer Research Trust.

### REFERENCES

Ciocca DR, Clark GM, Tandon AK, Fuqua SAW, Welch WJ and McGuire WL (1993) Heat shock protein hsp70 in patients with axillary lymph node-negative breast cancer: prognostic implications. *J Natl Cancer Inst* **85**: 570–574

Conroy SE and Latchman DS (1996) Do heat shock proteins have a role in breast cancer? *Br J Cancer* **74**: 717–721

Conroy SE, Faulds GB, Williams W, Latchman DS and Isenberg DA (1994) Detection of autoantibodies to the 90 kD heat shock protein in SLE and other autoimmune diseases. *Br J Rheumatol* **33**: 923–926

Conroy SE, Gibson SL, Brunstrom G, Isenberg D, Luqmani Y and Latchman DS (1995) Autoantibodies to the 90 kD heat shock protein in sera of breast cancer patients. *Lancet* **345**: 126–127

Conroy SE, Sassieni PD, Fentiman I and Latchman DS (1997) Autoantibodies to the 90 kD heat shock protein and poor survival in breast cancer patients. *Eur J Cancer* (press).

Creighton TE (1990) Protein folding. *Biochem J* **270**: 1–16

Dhillon VB, McCallum S, Norton PM, Twomey BM, Erkeller-Yuksel FM, Isenberg DA, Dhillon VB, Latchman DS and Lydyard PM (1992) Surface expression of heat shock protein 90 by blood mononuclear cells from patients with systemic lupus erythematosis. *J Autoimmunity* **5**: 803–814

Eskeller-Yuksel F, Lydyard P, Isenberg DA and Latchman DS (1993) Differential heat shock protein overexpression and its clinical relevance in systemic lupus erythematosis. *Ann Rheum Dis* **52**: 436–442

Faulds GB, Isenberg DA and Latchman DS (1994) The tissue-specific elevation in synthesis of the 90 kD heat shock protein precedes the onset of disease in lupus prone MRL/1pr mice. *J Rheumatol* **21**: 234–238

Faulds GB, Conroy SE, Madaeo M, Isenberg DA and Latchman DS (1995) Increased levels of autoantibodies to HSPs with increasing age in MRL/1pr mice. *Br J Rheumatol* 358–365

Ferrarini M, Heltai S, Zocchi MR and Rugarli C (1992) Unusual expression and localization of heat shock proteins in human tumor cells. *Int J Cancer* **51**: 613–619

Jameel A, Skilton RA, Campbell TA, Chandler SK, Coombes RC and Luqmani YA (1992) Clinical and biological significance of hsp90 alpha in human breast cancer. *Int J Cancer* **50**: 409–415

Jameel A, Law M, Coombes RC and Luqmani YA (1993) Significance of heat shock protein 90 as a prognostic indicator in breast cancer. *Int J Oncol* **2**: 1075–1080

© Cancer Research Campaign 1998

Konno A, Sato N, Yagihashi A, Torigoe T, Cho J, Torimoto K, Hara I, Wada Y, Okubo M, Takahashi and Kikuchi K (1989) Heat or stress inducible transformation associated cell surface antigen on the activated H-ras oncogene-transfected rat fibroblast. *Cancer Res* **49**: 6578–6582

Latchman DS (1991) Heat shock proteins and human disease. *J R Coll Phys* **25**: 295–300

Lindquist S (1986) The heat shock response. *Annu Rev of Biochem* **55**: 1151–1191

Lindquist S and Craig EA (1988) The heat shock proteins. *Annu Rev Gen* **22**: 631–677

Love S and King RJB (1994) A 27 kDa heat shock protein that has anomalous prognostic powers in early and advanced breast cancer. *Br J Cancer* **69**: 743–748

Lukacs KV, Lowrie DB, Stokes RW and Colston MJ (1993) Tumor cells transfected with a bacterial heat shock gene lose tumourigenicity and induce protection against tumours. *J Exp Med* 343–348

Lukacs KV, Nakakes A, Atkins CJ, Lowrie DB and Colston MJ (1997) In vivo gene therapy of malignant tumours with heat shock protein 65 gene. *Gene Ther* **4**: 346–350

Multhoff G, Botzler C, Wiesnet M, Maller E, Meier T and Wilmanns WA (1995) A stress inducible 72 kDa heat shock protein (hsp72) is expressed on the surface of human tumors but not on normal cells. *Int J Cancer* **61**: 272–279

Ritossa F (1962) A new puffing pattern induced and temperature shock and DNP in Drosophila. *Experientia* **18**: 571–573

Srivastava PK (1994) Peptide binding heat shock protein in the endoplasmic reticulum. Role in immune response to cancer and in antigen presentation. *Adv Cancer Res* **62**: 153–177

Srivastava PK and Heike M (1991) Tumor specific immunogenicity of stress induced proteins. Convergence of two evolutionary pathways of antigen presentation. *Sem Immunol* **3**: 57–66

Srivastava PK and Old LJ (1988) Individually distinct transplantation antigens of chemically induced mouse tumours. *Immunol Today* **9**: 78–83

Srivastava PK, De Leo AB and Old LJ (1986) Tumour rejection antigens of chemically induced sarcomas of inbred mice. *Proc Natl Acad Sci USA* **83**: 3407–3411

Thor A, Benz C, Moore D, Goldman E, Edgerton S, Landry J, Schwartz L, Mayall B, Hickey E and Weber LA (1991) Stress response protein (srp-27) determination in primary human breast carcinomas: clinical, histologic, and prognostic correlations. *J Natl Cancer Inst* **83**: 154–155

Tissieres A, Mitchell HK and Tracey UM (1974) Proteins synthesis in salivary glands of *D. melanogaster*. Relation to chromosome puffs. *J Mol Biol* **84**: 389–398

Tsuboi N, Ishikawa M, Tamura Y, Takayama S, Tobioka H, Matsuura A, Hirayoshi K, Nagata K, Sato N and Kikuchi K (1994) Monoclonal antibody specifically reacting against 73-kilodalton heat shock cognate protein: possible expression on mammalian cell surface. *Hybridoma* **13**: 373–381

Udono H and Srivastava PK (1993) Heat shock protein 70 associated peptides elicit specific cancer immunity. *J Exp Med* **178**: 1391–1396

Ullrich SJ, Robinson EA, Lain LW, Willingham M and Appella EA (1986) A mouse tumour specific transplantation antigen is a heat shock related protein. *Proc Natl Acad Sci USA* **10**: 3121–3125

© Cancer Research Campaign 1998